# Validation of the PILL-5: A 5-Item Patient Reported Outcome Measure for Pill Dysphagia

**DOI:** 10.3389/fsurg.2019.00043

**Published:** 2019-07-24

**Authors:** Nogah Nativ-Zeltzer, Ahmed Bayoumi, Van Pierre Mandin, Matthew Kaufman, Indulaxmi Seeni, Maggie A. Kuhn, Peter C. Belafsky

**Affiliations:** Department of Otolaryngology-Head and Neck Surgery, Center for Voice and Swallowing, University of California Davis School of Medicine, Sacramento, CA, United States

**Keywords:** pill dysphagia, questionnaire, tablet, outcome measure, swallowing

## Abstract

**Objectives:** Pill dysphagia is common and costly with a significant risk of pill retention, caustic injury, and poor medication compliance. The purpose of this investigation was to determine the validity and reliability of the PILL-5, a self-administered patient reported outcome measure (PROM) to quantify the degree of pill (tablet and capsule) dysphagia. The PILL-5 is a 5-item questionnaire with a maximum symptom score of 20.

**Methods:** The PILL-5 was administered to 190 patients with dysphagia referred for videofluoroscopic esophagography (VFE). Construct validity was assessed by comparing PILL-5 composite scores to delayed barium tablet transit on VFE. Normative data was obtained by administering the instrument to a cohort of healthy community based volunteers. Internal consistency was assessed with the Cronbach alpha. Test/retest reliability was determined by administering the instrument to the same cohort of patients at two time points.

**Results:** The mean PILL-5 was 5.6 (±4.9) for persons with dysphagia and 1.6 (±2.7) for healthy volunteers (*p* < 0.001). The internal consistency of the instrument was high (Cronbach alpha = 0.85). The mean PILL-5 was 4.3 (±4.1) for patients with normal transit and 7.6 (±5.3) for patients with delayed barium tablet transit on esophagography, indicating excellent criterion based validity (*p* < 0.001). Reproducibility was high with an intraclass correlation coefficient of 0.83 (*p* < 0.001).

**Conclusions:** Healthy individuals report some degree of swallowing difficulty with pills. Normative data suggest that a PILL-5 > 6 is abnormal (mean + 2 SD). The instrument demonstrated excellent criterion based validity and reliability. The PILL-5 is the first validated patient reported outcome measure for pill dysphagia.

## Introduction

Pill dysphagia, or difficulty swallowing tablets or capsules, is a common problem with significant health implications. Pill dysphagia can result from oropharyngeal sensory and motor deficits (1), esophageal strictures, webs, and rings, motility disorders (2), and phagophobia (3, 4). A 2003 survey of United States adults reported that ~40% of respondents had experienced difficulty swallowing pills (5). Pill dysphagia can result in pill retention, caustic injury (6, 7), pill aspiration (8, 9), and poor medication compliance (10, 11). To manage difficulty swallowing pills, alternate methods of drug delivery have been developed, including oral disintegrating tablets as well as liquid, nasal, pulmonary, and transdermal formulations (12). Patients and caregivers often modify tablets by splitting or crushing them. This practice can affect the biopharmaceutical features of medications and their effectiveness, creating a risk of adverse reaction (13, 14). Despite the high prevalence of pill dysphagia and its negative consequences, no self-administered patient reported outcome measure (PROM) for the severity of pill dysphagia currently exists. The purpose of this investigation was to determine the validity and reliability of the PILL-5, a 5-item self-administered patient PROM to quantify the degree of pill dysphagia.

## Methodology

The study was approved by the University of California, Davis Institutional Review Board (IRB). Written informed consent was obtained from all patient participants. A waiver of written consent was obtained from the UC Davis IRB for healthy participants, on the basis that the questionnaires were anonymous and could not be directly or indirectly identified. Verbal consent was obtained from these healthy individuals after providing them with a written information sheet per IRB protocol.

The PILL-5 is a 5-item Likert questionnaire with maximum (greatest symptoms) score of 20 (Table 1). The questions were originally derived from a list of 10 questions developed by expert consensus and obtained by the Delphi method, a systematic survey method to collect opinions from a panel of experts and reach consensus. The questions demonstrated high face validity and were abridged to a 5-item final version based on line-item redundancy. The questions address the localization of pill retention and the degree of disability (Table 1). Subjects were instructed to rate how frequently they experienced difficulty with swallowing pills by circling zero for never up to four for always.

**Table 1:** PILL-5 assessment tool.

Please circle the response that indicates how frequently you experience these symptoms.						
0 = Never; 1 = Almost Never; 2 = Sometimes; 3 = Almost Always; 4 = Always						
1.	Pills stick in my throat	0	1	2	3	4
2.	Pills stick in my chest	0	1	2	3	4
3.	I have a fear of swallowing pills	0	1	2	3	4
4.	My problem swallow pills interferes with my ability to take my medicine	0	1	2	3	4
5.	I can't take my pills without crushing, coating, or using other forms of assistance	0	1	2	3	4

The PILL-5 was administered to 190 patients with dysphagia referred for videofluoroscopic esophagography (VFE). Normative data was obtained by administering the instrument to a cohort (*n* = 226) of healthy community based volunteers. This healthy cohort had no history of swallowing dysfunction, gastroesophageal reflux, or other gastrointestinal disease, neurologic, or myopathic disease, or cancer of the head and neck, chest, or gastrointestinal tract.

Criterion-based validity of the instrument was assessed by comparing PILL-5 composite scores to delayed barium tablet transit on VFE. The VFE studies were completed in accordance with the standard protocol at our institution. Each subject was administered a bolus of liquid barium (EZ-PAQUE barium sulfate suspension, 60% w/v; 41% w/w, E-Z-EM, Inc., Westbury, NY) of 1, 20 mL in the lateral seated position and a 20 ml liquid barium bolus and 13 mm barium tablet (EZ Disk Barium Sulfate Tablet, Bracco Diagnostics, Monroe Township, NJ) in the AP standing position. Patients were then instructed to drink a 20 mL single swallow of barium followed by consecutive swallows of ~100 mL of barium from a cup sip in the right anterior oblique position. Finally, a water siphon test was performed in the supine position to evaluate for gastroesophageal reflux.

The anatomic structures and function of the pharyngoesophageal segment (PES) and esophagus were evaluated. Barium tablet transit was measured from the moment a verbal instruction to swallow was given until entry of the tablet into the stomach. Subjects were divided into groups of those with less than and those with more than 15 s of tablet transit. VFE recordings were analyzed by two independent raters blinded to the PILL-5 scores. A subset of 20 (10%) studies were analyzed by both raters in order to assess inter-rater reliability. PILL-5 composite scores of the normal and abnormal tablet transit groups were compared using the independent samples *t*-test.

In order to assess test-retest reproducibility of the instrument, the PILL-5 was administered to a subset of individuals (*N* = 74) on a second occasion at least 48 h and a maximum of 30 days after the initial administration. The intraclass correlation coefficient was determined and internal consistency was assessed with the Cronbach alpha.

## Results

### Reliability Assessment

The internal consistency of the instrument was high (Cronbach alpha = 0.85). Test-retest reliability was strong, with an intraclass correlation coefficient of 0.83 (*p* < 0.001).

### Normative Data

The mean (± SD) age of the healthy population (*n* = 226) was 49 (±18.6) years and 32% (*n* = 73) was male. The mean (±SD) age of the healthy participants aged 40 or above (*n* = 140) was 61.5 (±12.4) and 39% (*n* = 54) of the group was male (see Tables 2, 3 for patient characteristics).

**Table 2:** Distribution of patient diagnoses.

**Diagnostic category**	**Number of subjects**
Neurodegenerative/neuromuscular	8
Neurological Insult	2
Cervical spine abnormalities	12
Gastroesophageal Reflux Disease (GERD)	21
Esophageal dysmotility	26
Cricopharyngeal Muscle Dysfunction (CPMD)	84
Dysphagia-uncertain etiology	37

**Table 3:** Statistical summary of the PILL-5.

	**Q1**	**Q2**	**Q3**	**Q4**	**Q5**	**Total**
Patients with dysphagia	1.9 (±1.2)^*^	0.9 (±1.1)^*^	1.2 (±1.4)^*^	0.9 (±1.3)^*^	0.7 (±1.2)^*^	5.6 (±4.9)^*^
Normal cohort ≥40 years old	0.6 (±0.9)^*^	0.3 (±0.7)^*^	0.2 (±0.7)^*^	0.2 (±0.5)^*^	0.3 (±0.9)^*^	1.5 (±2.8)^*^
Normal cohort all ages	0.7 (±0.9)^*^	0.3 (±0.7)^*^	0.3 (±0.8)^*^	0.2 (±0.5)^*^	0.2 (±0.8)^*^	1.6 (±2.7)^*^

*Values are mean ± SD. Asterisk denotes statistically significant differences between patient and control cohorts*.

The mean (± SD) PILL-5 of the normal cohort was 1.6 (± 2.7) (Table 3). The data was normally distributed and a Pill 5 > 6 (Mean + 2 SD) represents the top 5% of responses.

### Construct Validity

The mean age of the patient population with dysphagia (*n* = 190) was 61.2 (±13.2) years and 42% (*n* = 79) was male. The mean total PILL-5 score for patients with dysphagia was 5.6 (±4.9). This was significantly higher than the PILL-5 of the normal population (*p* < 0.001) with a large effect size (Cohen's *d* = 1.03). The groups differed significantly on all items of the PILL-5 questionnaire (*p* < 0.001, Table 3). The mean (±SD) PILL-5 for patients with delayed barium tablet transit on esophagography was 7.58 (±5.3) in comparison to 4.3 (±4.1) for patients with normal barium tablet transit, indicating excellent criterion based validity (*p* < 0.001). The interrater reliability between the two clinicians assessing barium tablet transit times was high (κ = 0.86).

## Discussion

Pill dysphagia is common and costly (1, 10, 15). A recent survey suggests that four out of five adult Americans take several pills each day and nearly half report difficulty swallowing pills (11). Pill dysphagia is even more problematic in the elderly, who suffer a high prevalence of swallowing difficulty and are prescribed a large number of oral medications (16). Pill dysphagia can result in low medication adherence and treatment failure (17). In a survey of 540 nursing home residents, 15% of all inhabitants reported difficulty swallowing tablets and capsules. Of this group, 5% regularly expectorated their medication, while 27% refrained altogether from taking their medications (10). These findings are supported by the data from our investigation and suggest that some degree of baseline pill dysphagia is experienced by a large percentage of healthy adults.

Pill dysphagia can result in adverse outcomes. Stasis of tablets or capsules in the esophagus can affect the pharmacokinetics of medications and reduce effectiveness. Delayed transit may lead to premature drug-release, which reduces bioavailability and drug degradation (18). Moreover, pill retention can cause caustic injury due to prolonged contact of the medication with the esophageal mucosa (19–21). This can cause stricture formation, retrosternal pain, dysphagia, odynophagia, and in rare cases, can lead to complications such as mediastinal penetration, hemorrhage, and death (21). A recent report highlighted the dangers of the common recommendation of nil per os except medications given to patients with dysphagia, which puts patients at risk for pill aspiration and its consequences (8).

In recent decades, several global dysphagia self-assessment tools have emerged (22–24), however no self-administered assessment tool for pill dysphagia exists. Due to the unique clinical manifestations of pill dysphagia and the propensity for pill dysphagia to manifest without liquid or solid food dysphagia, there is a need for a tool focused specifically on defining pill dysphagia symptoms and its impact on patients. A recent investigation of persons admitted to an inpatient geriatric ward revealed that over 95% of patients with dysphagia were prescribed a solid formulation of medication that was potentially inappropriate for the degree of swallowing impairment with an average of nearly 2.5 inappropriate prescriptions per patient (25). A diagnostic tool specific for pill dysphagia can assist with safer and more efficacious prescribing habits. The advantage of a validated PROM over a simple binomial query of pill dysphagia (yes/no) is that the degree of swallowing impairment with pills can be quantified. This will allow clinicians to assess baseline pill dysphagia severity and alter their prescribing habits accordingly. In our experience, patients with minimal or no pill dysphagia (PILL-5 < 6) require no medication alteration, patients with mild to moderate pill dysphagia (PILL-5 ≥ 6 and < 12) can be managed with pill lubricants, and patients with moderate to severe pill dysphagia (PILL-5 ≥ 11) may require an altered formulation of medication. Appropriate referral to a swallowing specialist is warranted in individuals with a PILL-5 ≥ 6 to rule out obstructing pathology such an esophageal or cricopharyngeal stricture or web that may be easily treatable.

The data from this investigation suggest that the validity and reliability of the PILL-5 is high and that the instrument can differentiate between healthy controls and individuals with delayed barium tablet transit on swallowing fluoroscopy. Healthy volunteers without any history of swallowing difficulty reported a mean PILL-5 of 1.6 (±2.7). This suggests that some degree of difficulty swallowing pills is normal for most individuals. This is in contrast to dysphagia for solids and liquids, which are not typically experienced by healthy controls (23).

A limitation of the study is that the questionnaire was administered to patients who were referred for a VFE in a tertiary center. These patients may have clinical characteristics that are different from other patients with swallowing difficulty. Administration of the questionnaire to additional patient populations with and without dysphagia may demonstrate differences in subgroup PILL-5 scores. The cutoff of a PILL-5 ≥ 6 (mean + 2 SD) provides a conservative estimate for an abnormal instrument. Utilization of this cutoff may miss some patients with mild pathologic pill dysphagia.

## Conclusion

The PILL-5 is the first validated and reliable patient reported outcome measure for pill (capsule and tablet) dysphagia. Normative data suggest that a PILL-5 > 6 is significantly abnormal (mean +2 SD). The instrument demonstrated excellent criterion based validity and test-retest reliability.

## Ethics Statement

The study was approved by the University of California, Davis Institutional Review Board (IRB). Written informed consent was obtained from all patient participants.

## Author's Note

This work was presented at the American Bronchoesophagological Association during the Combined Otolaryngology Spring Meetings (COSM) on April 19, 2018, National Harbor, Maryland.

## Author Contributions

PB conceived the idea for the project, helped to collect the data, performed statistical analysis, and wrote and edited manuscript. NN-Z helped to collect the data, helped to perform the statistical analysis, and assisted with writing and editing the manuscript. VM, MK, and IS helped to collect the data. AB helped to collect the data and helped with the statistical analysis. MAK helped to edit the manuscript.

### Conflict of Interest Statement

The authors declare that the research was conducted in the absence of any commercial or financial relationships that could be construed as a potential conflict of interest.
